# Familiarity with words modulates interhemispheric interactions in visual word recognition

**DOI:** 10.3389/fpsyg.2022.892858

**Published:** 2022-07-29

**Authors:** Sangyub Kim, Joonwoo Kim, Kichun Nam

**Affiliations:** ^1^Wisdom Science Center, Korea University, Seoul, South Korea; ^2^School of Psychology, Korea University, Seoul, South Korea

**Keywords:** visual half-field study, bilateral redundancy gain, subjective familiarity, morphologically complex word, visual word recognition, interhemispheric interaction

## Abstract

Bilateral redundancy gain (BRG) indicates superior performance in bilaterally presented word recognition in the left and right visual fields (RVFs) relative to word recognition given in either the left or the RVF. The BRG may be modulated by participants’ subjective familiarity with words as previous studies found smaller regional activations in the brain as they become proficient. It can be assumed that visual recognition of words with high subjective familiarity indicates skilled performance in visual recognition. Thus, this study examined the subjective familiarity effect of visual words on the BRG during lateralized lexical decision performances. It showed that the significant BRG of response times was only observed in the most familiar word condition (F4 level); on the other hand, accuracy results revealed the significant BRGs in all the subjective familiarity levels (F1, F2, F3, and F4 levels). These results suggest that the bilateral presentation of identical words with higher subjective familiarity facilitates the recognition led by cooperative interactions between cerebral hemispheres. Therefore, the subjective familiarity with visual words modulates the efficiency of hemispheric interactions in visual word recognition.

## Highlights

- Superior performance was shown in the BVF words than in the UVF words.
- There was not significant bilateral redundancy gain in the pseudoword judgment.
- Familiarity with word modulates bilateral redundancy gain in visual word recognition.
- Familiar words lead to facilitation of interhemispheric interaction in visual recognition.

## Introduction

The hemispheric interaction between the left and the right hemispheres has been examined in various ways so far. The visual half-field study is one of the behavioral ways to investigate those hemispheric interactions in a normal population (Chiarello, 1988; Mohr et al., 1994). It propagates the visual stimuli to the visual cortical areas of the contralateral hemisphere for initial processing. The stimuli presented in the left visual field (LVF) are processed initially in the visual cortex of the right hemisphere and *vice versa*. This is because the human visual system allows the intersection between the visual field and the path of perceptual processing of the visual cortical regions (Bourne, 2006). Thus, by employing both of the visual fields as the presentation location, the visual half-field study has measured not only the lateralized responses of each hemisphere but also interactive responses between hemispheres.

Bilateral presentation (BVF) of identical words at the left and right visual fields (RVFs) is recognized faster and more accurately than words given in either visual field. This advantage has been known as “bilateral redundancy gain (BRG)” in visual word recognition studies (e.g., Mohr et al., 2007). BVF activates both hemispheres to process stimuli with accelerated interactions between cerebral hemispheres. Mohr et al. (1994) explained the BRG with the co-activation of the bilateral hemisphere from Hebb’s theory in cortical processing. They postulated that familiar or learned items have more interconnections of neural populations cortically connecting the two hemispheres (Pulvermüller and Mohr, 1996). Presentation of identical items in both visual fields would lead to greater activations in the bilateral hemisphere with stronger cortical representation resulting in superior performance in visual word recognition. Evidence of hemispheric interactions in the BVF of the normal population can be found in the visual word recognition studies using a lateralized lexical decision task (Abernethy and Coney, 1990; Mohr et al., 2007; Perrone-Bertolotti et al., 2013). For example, Mohr et al. (2007) compared the performances between the BVF and the unilateral presentation in the lateralized lexical decision task. They found significantly faster and more accurate responses of words in the BVF compared to the unilateral presentation, suggesting cooperative interactions between the two hemispheres in visual word recognition. In addition, a split-patient study with a divided visual field paradigm showed non-significant BRG, implicating that if there is no corpus callosum connecting the two hemispheres then it leads to a lack of hemispheric interactions triggered by the BVF (Mohr et al., 1994). The non-significant BRG in the split-patient study is induced by the disconnection of the corpus callosum which consists of 200∼300 million axonal projections between the bilateral hemisphere, meaning that the corpus callosum is a core connection for hemispheric interactions (Nowicka and Tacikowski, 2011). These previous studies implicate that bilateral word presentation in the unilateral visual fields certainly leads to hemispheric interactions for visual word recognition.

However, the strength of the hemispheric interactions may be dependent on the degree of familiarity with the items. As explained by the co-activation of Hebb’s theory in cortical processing (Mohr et al., 1994), the extent to which an item is familiar or learned determines the cortical representation of both hemispheres, which indicates that high familiarity words are more largely represented with stronger interconnections of neural assemblies distributed in the bilateral hemisphere. Hence, the neuronal summation mechanism that leads to a stronger representation in the BVF of identical words can be modulated by the familiarity of those words. This is because higher familiarity assumes to show more proficient processing within and/or between hemispheres for required processes, such as visual word recognition (Connine et al., 1990), and familiarity decision of faces (Mohr et al., 2002). According to a bunch of previous studies, the benefits of familiarity in learning were established through repetitive experience (e.g., Ebbinghaus, 1913; Hintzman, 1976), suggesting that familiarity was led by repetition and induced dynamic hemispheric interaction.

The current study hypothesized that the subjectively rated familiarity with words modulates the BRG in visual word recognition, showing a greater gain of the bilateral redundancy in the recognition of words with higher subjective familiarity. Therefore, this study aims to investigate the subjective familiarity effect of words on the BRG during the lateralized lexical decision by comparing the performances in the BVF and the unilateral presentation according to subjective familiarity with words.

## Materials and methods

### Participants

A total of 37 participants took part in the experiment. One participant who failed to comply with the experimental procedures was excluded from the response times and accuracy analysis. Hence, data from 36 participants were analyzed (19 women; 23.84 ± 2.39 years, *M* ± *SD*). All the participants were strongly right-handed (*M*: 8.05 points, *SD*: 1.82) as evaluated by the Edinburgh Handedness Inventory (Oldfield, 1971). All the participants had normal or corrected-to-normal vision of both eyes and had no medical history of neurological impairment. This study was approved by the ethics committee of the Korea University, South Korea, where the current study was performed. In addition, this study complied with the ethical standards laid down in the 1964 Declaration of Helsinki. All participants understood the code of ethics and gave informed consent before participation.

### Experimental task

Lateralized lexical decision is a traditional way to examine the lexical processing of unilateral and bilateral hemispheres (i.e., Mohr et al., 1994, 2002, 2007; Kim et al., 2020). It leads to initial activations in the contralateral hemisphere corresponding to the unilateral visual field in which stimuli is presented, thus enabling to measure the lateralized responses of a hemisphere (Mohr et al., 2002). In addition, the association between BVF in both visual fields and interhemispheric interaction has been consistently reported (Mohr et al., 2002, 2007; Chu and Meltzer, 2019). It is useful experimental manipulation for hemispheric investigation even though unilateral presentation finally leads to activation of the contralateral hemisphere but also the other hemisphere as time goes by Barca et al. (2011). Participants were instructed to judge whether visual letter strings presented in the LVF, RVF, or bilateral visual field (BVF) of the screen were a word or a pseudoword while they were fixating their eyes on the fixation point (“+”) in the middle of the screen. The pseudowords were employed for filler word conditions having no meaning but were orthographically legal and pronounceable. The order of the stimuli was randomized. Responses of participants were made by pressing the “slash (/)” for word judgment or the “*Z*” button for pseudoword judgment on the keyboard with the index finger of each hand. The response hands were counterbalanced among participants. Participants were instructed to judge as fast and as accurately as possible and to fixate their eyes on the fixation point. The task measured how fast the participant respond correctly to a word (word response time) and to a pseudoword (pseudoword response time) and how accurately the participant respond to a word (word accuracy) and a pseudoword (pseudoword accuracy).

### Experimental procedure

**FIGURE 1:**
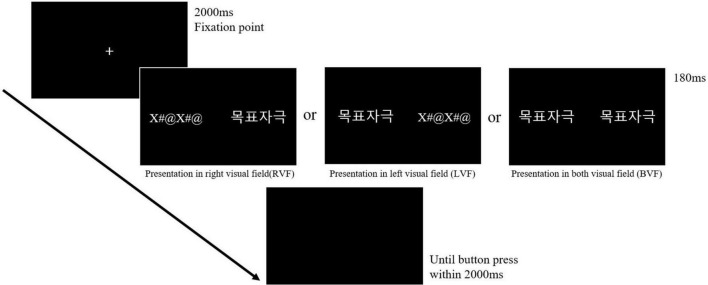
Experimental paradigm of the lateralized lexical decision task. The fixation point was presented for 2,000 ms to make subjects fixate in the middle of the screen. Then, the stimuli were presented in the RVF, LVF, or BVF with the presentation of the string of symbols (“X#@X#@”) in the opposite visual field in the case of the RVF and the LVF. Subjects were required to make a lexical decision after the disappearance of stimuli within 2,000 ms to proceed next trial. This figure presents the location of the target at each visual field with “목표자극(target)” in Korean.

The fixation point was presented in the center of the screen for 2,000 ms, followed by a stimulus given in the left, right, or both visual fields of the screen for 180 ms. The short presentation of the stimuli for 180 ms in the parafoveal vision was due to avoidance of gaze shifting toward the unilateral visual field. In the unilateral presentation, a string of symbols (“X#@X#@”) was simultaneously presented in the opposite visual field of the stimuli presentation. The participants had to decide whether the stimulus was a word or not within 2,000 ms during a black empty screen presented after the deletion of the target. The schematic illustration of the experimental procedure is shown in Figure 1. About 12 practice trials were presented before 600 main trials (300 words and 300 pseudowords) began. All stimuli in the main trials were shown in the pseudo-randomized order and presented only once during the experiment. Moreover, three stimuli lists were made by the Latin-square design to give each stimulus to all visual field conditions (RVF, LVF, and BVF). Each stimulus set consisted of 300 words and 300 pseudowords, and each participant was assigned to one of the lists (list 1, list 2, or list 3) so that each 12 out of 36 participants performed the assigned stimuli list.

### Apparatus

The RGB-colored LG monitor was used to give stimuli in the experimental room. The distance between the participant (nasion of the face) and the screen was kept at 65 cm. All the stimuli were white letters and presented within 2°˜5° horizontal and 1.5° vertical visual angles on a black background. The stimulus presentation and duration were controlled by E-prime 2.0 professional (Psychology Software Tools, Inc., Pittsburgh, PA, United States). All the participants kept their chin on a chin rest during the experiment with their forehead staying in a centered stationary bar to fix their gazing point. A keyboard was placed in front of the participants and serves to collect the participants’ responses.

### Materials

About 300 morphologically complex words were randomly extracted from movies (10%), newspapers (20%), books (30%), and Internet blogs or posts (40%; Kim et al., 2020). These extracted words are ecologically valid stimuli since they are not biased in specific purposes of experiments. In addition, 300 Korean pseudowords were constructed by randomly combining the syllables used in the extracted words to not be defined in the Korean Sejong Corpus of Kang and Kim (2009). The pseudowords were orthographically legal and pronounceable but had no meanings.

### Experimental conditions

This study used previously surveyed data of subjective familiarity on 300 words (Kim et al., 2020). They asked participants to rate how familiar each word was on a seven-point scale. A score of one indicates the most unfamiliar word, whereas a score of seven indicates the most familiar word. Since this study targeted the subjective familiarity effect of words on the BRG, the subjective familiarity of words was divided into four levels based on the measured score, that is, F1 level (the most unfamiliar words; *M* = 4.21, *SD* = 0.74), F2 level (slightly unfamiliar word; *M* = 4.61, *SD* = 0.91), F3 level (slightly familiar word; *M* = 5.10, *SD* = 0.75), and F4 level (the most familiar word; *M* = 5.46, *SD* = 0.78). There was a significant difference in subjective familiarity between those four levels [*F*(3, 296) = 35.415, *p* < 0.001], and the Bonferroni *post hoc* test revealed that there was a significant gradual increase in scores from F1 level to F4 level subjective familiarity (*p* < *0.001* for the F1 score < the F2 score; *p* = *0.272* for the F1 score < the F3 score; *p* < *0.001* for the F1 score < the F4 score; *p* < *0.001* for the F2 score < the F3 score; *p* = *0.167* for the F2 score < the F4 score; *p* < *0.001* for the F3 score < the F4 score). In addition, other seven lexical variables (number of strokes, number of phonemes, number of syllables, number of morphemes, number of objective meanings, and frequency of the first syllable) potentially influencing the visual word recognition are described in Tables 1, 2 (Kim et al., 2020). Those six lexical variables were statistically similar between the four subjective familiarity levels as described in Table 3. The values of the six lexical variables were evaluated by Korean Sejong Corpus (Kang and Kim, 2009).

**TABLE 1:** Description of the lexical variables in experiment of this study.

Lexical variable	Content	Value
# of syllables	‘개(gae)+념(nyum)+을(eoul)	3
# of morphemes	‘개념(gae-nyum: root)+을(eoul: affix)	2
# of phonemes	ㄱ+ㅐ+ㄴ+ㅕ+ㅁ+ㅇ+ㅡ+ㄹ	8
First syllable frequency	Number of Eojeols using the same first syllable(“‘개(gae)”)	8,963
# of objective meanings	Number of dictionary meanings of “‘개념을”	1
# of strokes	Number of strokes of “‘개념을”	21

The example morphologically complex word “‘개념을(gae-nyum-eoul)” is presented for explaining the attributes of the lexical variables.

**TABLE 2:** Descriptive statistics of the lexical variables of stimuli in experiment.

Lexical variable	Range	Mean	Standard deviation	Value
# of syllables	2–4	3.20	0.53	300
# of morphemes	1.66–4	2.09	0.31	300
# of phonemes	4–12	8.06	1.55	300
First syllable frequency	1.86–4.42 (log)	3.73 (log)	0.49 (log)	300
# of objective meanings	1–14	1.5	1.23	300
# of strokes	8–33	18.83	4.53	300

**TABLE 3:** Descriptive statistics of each level in subjective familiarity condition.

	Length variable				Semantic variable	Frequency variable	
	Number of strokes	Number of phonemes	Number of syllables	Number of morphemes	Number of objective meanings	First syllable frequency	Subjective familiarity
F1 (the most unfamiliar word)	19.43 (4.92)	8.21 (1.51)	3.29 (0.49)	2.10 (0.34)	1.35 (0.69)	3.76 (0.48)	4.21 (0.74)
F2 (slightly unfamiliar word)	18.96 (4.46)	8.05 (1.64)	3.24 (0.057)	2.08 (0.30)	1.65 (1.80)	3.68 (0.58)	4.61 (0.91)
F3 (slightly familiar word)	19.19 (4.10)	8.09 (1.31)	3.19 (0.46)	2.12 (0.33)	1.49 (1.07)	3.75 (0.41)	5.10 (0.75)
F4 (the most familiar word)	17.76 (4.53)	7.89 (1.74)	3.09 (0.60)	2.09 (0.29)	1.51 (1.10)	3.73 (0.87)	5.46 (0.78)

The values within brackets denote the standard error.

## Results

**FIGURE 2:**
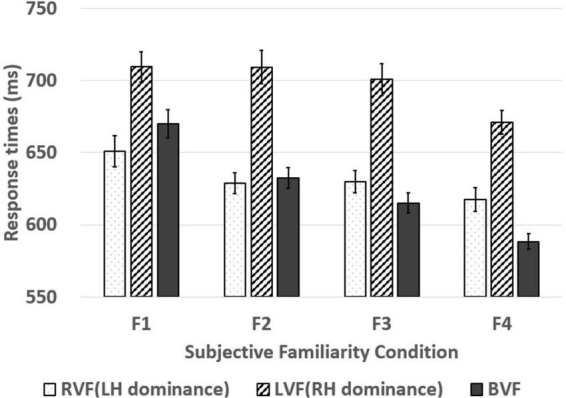
Response times of words presented in the RVF, LVF, and BVF in four subjective familiarity levels. The four subjective familiarity levels indicate F1 (*M* = 4.21, *SD* = 0.74), F2 (*M* = 4.61, *SD* = 0.91), F3 (*M* = 5.10, *SD* = 0.75), and F4 (*M* = 5.46, *SD* = 0.78). The line in the bar graph denotes the range of standard error.

**FIGURE 3:**
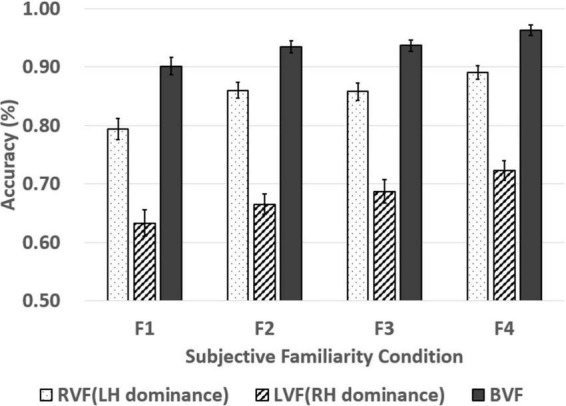
Accuracy of words presented in the RVF, LVF, and BVF in four subjective familiarity levels. The four subjective familiarity levels indicate F1 (*M* = 4.21, *SD* = 0.74), F2 (*M* = 4.61, *SD* = 0.91), F3 (*M* = 5.10, *SD* = 0.75), and F4 (*M* = 5.46, *SD* = 0.78). The line in the bar graph denotes the range of standard error.

We performed statistical analyses based on item analyses due to investigation of the subjective familiarity effect of words in lateralized word recognition. The results of the lateralized lexical decision depending on the subjective familiarity of words are described in Table 4 and Figures 2, 3. The BRG was measured in superior performance in the BVF relative to the best performance among unilateral presentations (RVF/LVF; Mohr et al., 2007). This study found a superior performance in the RVF compared to the LVF in all four subjective familiarity conditions for response times and accuracy as paired *t*-test showed significantly higher accuracy and faster responses in the RVF presentation than the LVF presentation (see Table 5). This indicates that the performance of the RVF should be compared to the BVF to measure the BRG.

**TABLE 4:** Results of response times (RTs) and accuracy (ACC) at each visual field (RVF, LVF, and BVF) were shown according to the level of subjective familiarity with words.

		RVF (LH dominance)		LVF (RH dominance)		BVF	
		RTs	ACC	RTs	ACC	RTs	ACC
Subjective familiarity levels	F1	651 (11)	0.79 (0.02)	709 (11)	0.63 (0.02)	670 (10)	0.90 (0.02)
	F2	629 (7)	0.86 (0.01)	709 (12)	0.67 (0.02)	632 (7)	0.93 (0.01)
	F3	630 (8)	0.86 (0.01)	701 (11)	0.69 (0.02)	615 (7)	0.94 (0.01)
	F4	618 (8)	0.89 (0.01)	671 (8)	0.72 (0.02)	588 (5)	0.96 (0.01)

The values within brackets denote standard error. Four subjective familiarity levels, F1 (*M* = 4.21, *SD* = 0.74), F2 (*M* = 4.61, *SD* = 0.91), F3 (*M* = 5.10, *SD* = 0.75), and F4 (*M* = 5.46, *SD* = 0.78).

**TABLE 5:** Results of paired *t*-test (RVF vs. LVF) at each subjective familiarity level in response times (RTs) and accuracy (ACC) for words

		RTs	ACC
Subjective familiarity levels	F1	RVF < LVF (ms) *t*(74) = -4.371, *p* < *0.001*	RVF > LVF (%) *t*(74) = 6.715, *p* < *0.001*
	F2	RVF < LVF (ms) *t*(74) = -6.373, *p* < *0.001*	RVF > LVF (%) *t*(74) = 9.877, *p* < *0.001*
	F3	RVF < LVF (ms) *t*(74) = -5.792, *p* < *0.001*	RVF > LVF (%) *t*(74) = 8.579, *p* < *0.001*
	F4	RVF < LVF (ms) *t*(74) = -5.418, *p* < *0.001*	RVF > LVF (%) *t*(74) = 9.584, *p* < *0.001*

For response times, subjective familiarity (four levels: F1/F2/F3/F4) × visual field (three levels: RVF/LVF/BVF) two-way repeated-measures analyses of variance (ANOVAs) were conducted. The main effects of the subjective familiarity and the visual field were significant [*F*(3, 222) = 11.877, *p* < 0.001, $\eta_{p}^{2}$ = 0.138; *F*(2, 148) = 120.540, *p* < 0.001, $\eta_{p}^{2}$ = 0.620]. Also, the two-way interaction was significant [*F*(6, 444) = 2.790, *p* = 0.011, $\eta_{p}^{2}$ = 0.036]. The Bonferroni *post hoc* test for the main effect of the subjective familiarity revealed faster response times in lexical decisions on words with higher subjective familiarity (*p* = 0.024 for F1-F2; *p* = 0.257 for F1-F3; *p* < 0.001 for F1-F4; *p* = 0.999 for F2-F3; *p* = 0.010 for F2-F4; *p* = 0.002 for F3-F4). In addition, the Bonferroni *post hoc* test for the main effect of the visual field showed significant differences between RVF and LVF (*p* < 0.001) and between BVF and LVF (*p* < 0.001), whereas it did not show a significant difference between RVF and BVF (*p* = 0.652). A simple main effect analysis on the two-way interaction effect showed the main effect of the visual field in all subjective familiarity conditions [*F*(2, 148) = 12.548, *p* < 0.001, $\eta_{p}^{2}$ = 0.145 for F1; *F*(2, 148) = 34.036, *p* < 0.001, $\eta_{p}^{2}$ = 0.315 for F2; *F*(2, 148) = 32.746, *p* < 0.001, $\eta_{p}^{2}$ = 0.307 for F3; *F*(2, 148) = 38.867, *p* < 0.001, $\eta_{p}^{2}$ = 0.344 for F4]. However, the Bonferroni *post hoc* test for the main effect of the visual field only showed a significant difference between RVF and BVF in F4 subjective familiarity condition (*p* = 0.271 for F1; *p* = 0.350 for F2; *p* = 0.999 for F3; *p* = 0.003 for F4), indicating only significant BRG in F4 subjective familiarity condition.

**FIGURE 4:**
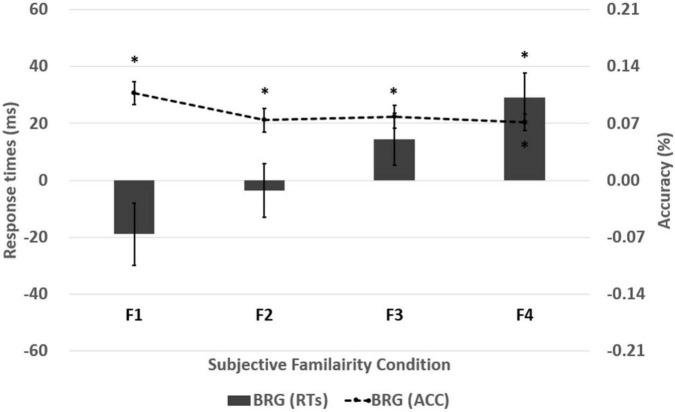
The BRGs were obtained from the four subjective familiarity levels. The four subjective familiarity levels indicate F1 (*M* = 4.21, *SD* = 0.74), F2 (*M* = 4.61, *SD* = 0.91), F3 (*M* = 5.10, *SD* = 0.75), and F4 (*M* = 5.46, *SD* = 0.78). The line in the bar graph denotes the range of standard error. The asterisk indicates the significance of effect in one-sample *t*-test in response times and accuracy (*p < 0.05).

Additionally, subjective familiarity (four levels: F1/F2/F3/F4) × visual field (three levels: RVF/LVF/BVF) two-way repeated-measures ANOVAs were conducted on the accuracy. The main effects of the subjective familiarity and the visual field were significant [*F*(3, 222) = 8.375, *p* < 0.001, $\eta_{p}^{2}$ = 0.102; *F*(2, 148) = 427.229, *p* < 0.001, $\eta_{p}^{2}$ = 0.852]. However, the two-way interaction was not significant [*F*(6, 444) = 0.758, *p* = 0.603, $\eta_{p}^{2}$ = 0.010]. The Bonferroni *post hoc* test for the main effect of the subjective familiarity revealed more accurate responses in lexical decisions on words with higher subjective familiarity (*p* = 0.005 for F1-F2; *p* = 0.106 for F1-F3; *p* < 0.001 for F1-F4; *p* = 0.999 for F2-F3; *p* = 0.311 for F2-F4; *p* = 0.030 for F3-F4). In addition, the Bonferroni *post hoc* test for the main effect of the visual field showed more accurate responses in BVF than RVF and LVF (*p* < 0.001; *p* < 0.001) and more accurate responses in RVF than LVF (*p* < 0.001). It indicates a significant BRG in all the subjective familiarity conditions. All the results of the BRGs in response times and accuracy are described in Table 6 and Figure 4.

**TABLE 6:** Results of the BRGs at each subjective familiarity level in response times (RTs) and accuracy (ACC) for words.

		Bilateral redundancy gain (BRG)	
		RTs	ACC
Subjective familiarity levels	F1	-18.89 (11.01)	0.11 (0.01)
	F2	-3.59 (9.41)	0.07 (0.01)
	F3	14.52 (9.14)	0.08 (0.01)
	F4	29.25 (8.52)	0.07 (0.01)

BRG in RTs: RVF response – BVF response, BRG in ACC: BVF response – RVF response. The values within brackets denote the standard error.

**FIGURE 5:**
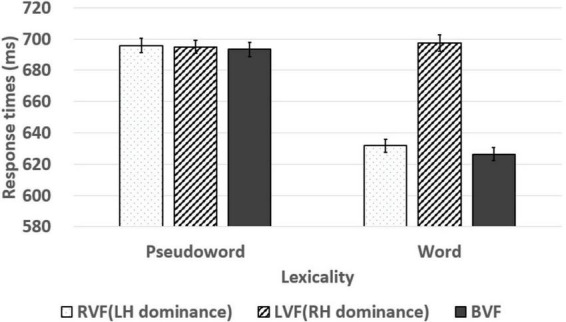
Response times of pseudoword and word at the RVF, LVF, and BVF. The line in the bar graph denotes the range of standard error.

**FIGURE 6:**
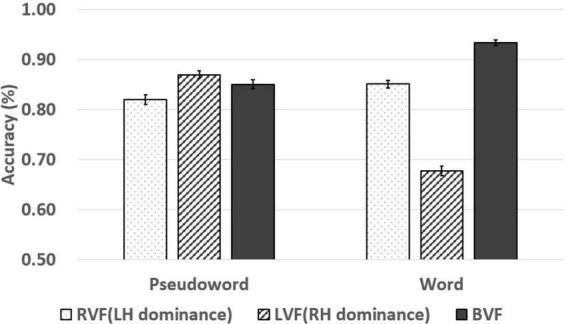
Accuracy of pseudoword and word at the RVF, LVF, and BVF. The line in the bar graph denotes the range of standard error.

In addition, the results of the pseudoword and the word judgments in the lateralized lexical decision task are shown in Table 7 and Figures 5, 6. Lexicality (two levels: word/pseudoword) × visual field (three levels: RVF/LVF/BVF) two-way repeated-measures ANOVAs were conducted on the response times. The main effects of the lexicality and the visual field were significant [*F*(1, 299) = 88.803, *p* < *0.001*, $\eta_{p}^{2}$ = 0.229; *F*(2, 598) = 52.958, *p* < *0.001*, $\eta_{p}^{2}$ = 0.150]. Also, the two-way interaction was significant [*F*(2, 598) = 52.908, *p* < *0.001*, $\eta_{p}^{2}$ = 0.150]. The main effect of the lexicality indicates faster responses in the word judgment than the pseudoword. Furthermore, the main effect of the visual field denotes that the responses in the RVF and the BVF were faster than in the LVF with no difference between response times in the RVF and the BVF. Simple main effect analysis on the two-way interaction effect revealed significantly faster responses in the RVF and the BVF than in the LVF with no difference between the responses in the RVF and the BVF in the word judgment (*p* < *0.001*; *p* < *0.001*). However, there were no significant differences between the response times in each visual field (RVF/LVF/BVF) in the pseudoword judgments (*p* = *0.858* for the RVF and the LVF comparison; *p* = *0.676* for the RVF and the BVF comparison; *p* = 756 for the LVF and the BVF comparison). Lexicality (two levels: word/pseudoword) × visual field (three levels: RVF/LVF/BVF) two-way repeated-measures ANOVAs were conducted on the accuracy rates. The main effects of the lexicality and the visual field were significant [*F*(1, 299) = 7.916, *p* = *0.005*, $\eta_{p}^{2}$ = 0.026; *F*(2, 598) = 196.092, *p* < *0.001*, $\eta_{p}^{2}$ = 0.396]. Also, the two-way interaction was significant [*F*(2, 598) = 299.604, *p* < *0.001*, $\eta_{p}^{2}$ = 0.501]. The main effect of the lexicality indicates more accurate responses in the pseudoword than in the word. And, The Bonferroni *post hoc* test for the main effect of the visual field denotes that the more accurate responses in the BVF than the LVF and the RVF (*p* < *0.001*; *p* < *0.001*), and more accurate responses in the RVF than the LVF (*p* < *0.001*). Simple main effect analysis on the two-way interaction effect showed the main effects of the visual field in the pseudoword judgment [*F*(2, 598) = 18.911, *p* < *0.001*, $\eta_{p}^{2}$ = 0.59] and in the word judgment [*F*(2, 598) = 444.612, *p* < 0.001, $\eta_{p}^{2}$ = 0.598]. The Bonferroni *post hoc* test for the main effect of the visual field revealed more accurate responses in the BVF than the LVF and the RVF (*p* < 0.001; *p* < 0.001), and more accurate responses in the RVF than the LVF (*p* < *0.001*) in word judgment. In addition, the Bonferroni *post hoc* test for the main effect of the visual field showed less accurate responses in the RVF than the LVF and the BVF (*p* < *0.001*; *p* = *0.002*), and more accurate responses in the LVF than the BVF (*p* = *0.031*).

**TABLE 7:** Response times (RTs) and accuracy (ACC) at the RVF, LVF, and BVF for words and pseudowords were described.

	RVF (LH dominance)		LVF (RH dominance)		BVF	
	
	RTs	ACC	RTs	ACC	RTs	ACC
Pseudoword	696 (5)	0.82 (0.01)	695 (4)	0.87 (0.01)	693 (5)	0.85 (0.01)
Word	632 (4)	0.85 (0.01)	697 (5)	0.68 (0.01)	626 (4)	0.93 (0.01)

The values within brackets denote standard error.

## Discussion

This study revealed the subjective familiarity effect of words on the BRG when the words are visually recognized. In response times analysis, there were no significant BRGs in the F1, the F2, and the F3 subjective familiarity levels except the F4 subjective familiarity level, strongly supporting the hypothesis of the greater BRG in the visual recognition of words with higher subjective familiarity. On the other hand, in accuracy, the significant BRG was steadily observed in the four subjective familiarity levels (F1, F2, F3, and F4). The results in response times showed a different pattern unlike accuracy as the significant BRG of response times was only observed in the F4 subjective familiarity level. It suggests that the interhemispheric interaction facilitates visual recognition when the familiar word is presented bilaterally, whereas the unfamiliar word rather leads to uncooperative interaction between the two hemispheres in bilaterally presented word recognition.

The current study supports the idea that the way of interhemispheric interactions (facilitation or inhibition) between cerebral hemispheres depends on the familiarity of items, in particular words. In this regard, there have been previous studies that addressed changes of functional activations as developing proficiency even though these studies differ from word-related investigations, which enables us to infer the mechanism of interhemispheric interactions in a process of being proficient. Qin et al. (2003) observed the brain activations with functional magnetic resonance imaging that a prefrontal region showed gradually decreased activations in the practice of artificial algebra learning but activations of a parietal region were less affected by the practice. It suggests that being more cognitively familiar with the algebra calculation accompanies changes in interactions of activated regions, largely requiring the parietal region involved in imagined transformations and/or the motor regions concerned with manual programming. It resembles the findings of the current study as we observed the cooperative interactions between the two hemispheres in the most familiar word condition that is assumed to have a lot of practice experience relative to the unfamiliar word condition. In addition, Hill and Schneider (2006) reported the negative relationship between the regional activations in the brain and the degree of skillful performance in the motor tracking task. They found more activations in the frontal, motor, and parietal regions in the novice group but the frontal and the parietal activations were diminished in the skilled group with almost remaining activations in the motor area for performances in the motor tracking task. It implicates that proficiency may be closely associated with the degree of interactions between activational regions since they found smaller activation regions in the skilled group relative to the novice group. The involvement of smaller brain regions in proficient processing occurred by regional interactions to process more efficiently. This is because the brain decided where should be activated to process and mostly the activation regions are associated with the performances that a task requires. For example, the parietal region functions as the main part to perform the motor tracking task in Hill and Schneider (2006), since the authors discovered the remaining activations in the parietal regions only in the skilled group, but not in the unskilled group. The regional cooperative interactions were required to leave a few regions mainly involved in the processing and to show more efficient participation with minimal regions by reducing the unnecessary metabolism in the brain. However, at the early stage of proficiency, hemispheres may exhibit considerably competitive responses by independent participation of each activational region rather than cooperative interaction. If we compare this explanation to the results of the current study, the finding of significant BRG only in the greatest familiar word condition was able to explain the cooperative mechanism of interhemispheric interactions, which shows the most efficient processing of the two hemispheres. In addition, the non-significant BRG in the pseudoword judgment indicates a lack of cooperative interactions between the two hemispheres, meaning the early stage of proficiency in word processing. The current study enables us to infer the interhemispheric interactions of being proficient since the hemispheric response for proficiency is expected to follow a similar pattern in a diminished mechanism as shown in Qin et al. (2003) and Hill and Schneider (2006) even though their cognitive domains were distant.

On the one hand, the results of faster responses in the RVF presentation compared to the LVF presentation were not observed in the pseudoword judgment, suggesting not dominantly processed by the left hemisphere but rather requires independent processing of each hemisphere. These results are consistent with the previous studies reporting non-significant BRG and a right visual field advantage (RVFA) which indicates superior performances of the RVF than the LVF in word judgments during the lateralized lexical decision task in contrast with pseudoword judgments (e.g., Young et al., 1980; Bradshaw and Nettleton, 1983; Hellige, 1993; Mohr et al., 2007). Mohr et al. (2007) showed the non-significant BRG and RVFA in response times and accuracy of the pseudoword judgment in contrast with the word judgment in the lateralized lexical decision task. They also found neurophysiological evidence with ERPs (event-related potentials) that a significant increase of amplitude 160–200 ms after the BVF relative to the unilateral presentation specifically in the word judgments. Their source localized analysis using minimum norm estimation revealed greater cortical activation of word recognition in temporal regions of the left and the right hemispheres after the BVF relative to each of the unilateral presentations. On the other hand, there was not such a significant increase in cortical activity in the pseudoword judgment. In addition, significant advantages in word presentations given in the RVF compared to the LVF (RVFA) imply the left hemisphere’s dominance for language processing since the RVF initially gives the stimuli to the left hemisphere (Knecht et al., 2000). Conversely, the LVF initially projects to the right hemisphere leading to the transfer toward the left hemisphere for word processing through corpus callosum or subcortical areas (Nowicka and Tacikowski, 2011), which consequently makes word recognition slower and inaccurate. However, the pseudoword processing may be not required of them as the significant RVFA was not found in the pseudoword judgment, implying that the left hemisphere is not the dominant hemisphere for the pseudoword processing assuming independent processing of each hemisphere. In this regard, there have been studies reporting the significance of the right hemisphere in visual object recognition (Hellige and Webster, 1979; Warrington and James, 1986), which is considered as a work of the right hemisphere to recognize unfamiliar objects, including the pseudoword judgments. Indeed, pseudowords and words with low subjective familiarity have in common the sense of personal familiarity on them. Since both the pseudowords and the words with low subjective familiarity are unfamiliar, they may be expected to show a rather similar pattern of hemispheric interaction in processing. Of course, they are not identically unfamiliar as words with low subjective familiarity are still words that are stored in the mental lexicon. However, the point is “unfamiliarity” itself with items. Personal unfamiliarity (and familiarity) is dependent on their experience. The level of the unfamiliarity of items gradually decreases as we experience the items repeatedly, while the familiarity level increases after repetitive experiences. This is why both the pseudoword and the low familiar word would be interpretable in a continuous scale of familiarity, and it will show a consistent tendency to generalize the visual processing depending on the subjective familiarity of items. Thus, recognition of bilaterally presented unfamiliar words is assumed to show independent processing of the two hemispheres, which may be able to lead to competitive interaction between the two hemispheres in visual word processing.

Previous studies have provided two possible mechanisms of bihemispheric processing for collaboration in word processing. They suggested the metacontrol model (Levy and Trevarthen, 1976; Hellige, 1993), and the cooperative model (Miller, 1982; Allen, 1983; Mordkoff and Yantis, 1991). The metacontrol model indicates that one hemisphere dominantly takes charge of the processing over the other hemisphere, i.e., the dominant processing of the left hemisphere in lexical processing. And, the cooperative model denotes the cooperation of two hemispheres in the processing. The findings of the current study support the cooperative model in visual word recognition as we found the significant BRG in the greatest familiar condition, which replicates the previous reports (i.e., Yoshizaki, 2001). If the two hemispheres follow the metacontrol model, the BRG would not be significantly observed in the greatest familiar condition in this study since the left hemisphere is mainly responsible for word processing. In addition, the current study specifically contributes to the understanding of how two halves of the brain interact in a process of being proficient. This study found the significant changes in the BRG according to a gradual increase from the lowest familiar condition to the greatest familiar condition. It is convincing evidence to explain why our two hemispheres show efficient processing if we are excellent at a particular task. It is because the two hemispheres interact with each other to cooperate for more efficient processing, which reduces unnecessary hemispheric processing. Cooperation is one of the keys to explaining the mechanism of being proficient in word processing, even though it may work on other domains of cognitive processing, such as memory and attention. Therefore, the current study contributes to understanding the general mechanism of interhemispheric interaction in a process of being proficient in cognitive processing, in particular, in the cognitive domain of visual word processing.

Therefore, there are two implications of this study. The first implication of the current study was a clear demonstration of the mechanism of hemispheric interaction in terms of familiarity. We were able to assume high proficiency in the processing of words with high familiarity and low proficiency in the processing of words with low familiarity. We quantitatively measured subjective familiarity with words by self-report so that we divided the familiarity condition into four categories, which is a more precise way to examine the effect of familiarity on the interhemispheric interaction than the previous studies, such as Yoshizaki and Hatta (2005). The only significance of the bilateral gain at the greatest familiar word condition in the current study suggests the significant mediation of familiarity on the cooperative interaction between the two hemispheres. The second is to examine interhemispheric interaction in word processing using word and pseudoword stimuli. For example, subjects in the study of Yoshizaki and Hatta (2005) performed alphabet-matching task, which relies more on memory rather than word processing. Yoshizaki and Hatta (2005) found the benefits of bihemispheric processing as the amount of learning increased, which is in line with the findings of the current study albeit it used the lateralized lexical decision task for the investigation of interhemispheric interaction. It contributes to generalizing the findings of such previous studies, i.e., Yoshizaki and Hatta (2005).

Consequently, the current study found an increase in the BRG in the visual recognition of words with higher subjective familiarity. It suggests changes in the interhemispheric interactions during recognition of a higher subjective familiarity word from rather independent processing within the hemisphere to cooperative participation between the two hemispheres.

## Data availability statement

The original contributions presented in this study are included in the article/supplementary material, further inquiries can be directed to the corresponding author/s.

## Ethics statement

The studies involving human participants were reviewed and approved by Korea University Institutional Review Board. The patients/participants provided their written informed consent to participate in this study.

## Author contributions

SK: conceptualization, methodology, investigation, formal analysis, project administration, visualization, and writing—original draft, review and editing. JK: project administration and writing—original draft. KN: conceptualization and supervision. All authors contributed to the article and approved the submitted version.
